# Whole genome-based characterization of virulence and antimicrobial resistance determinants in clinical *Campylobacter jejuni* isolates from Minnesota, 2018–2021

**DOI:** 10.1128/jcm.00514-26

**Published:** 2026-10-27

**Authors:** Haejin Hwang, Melanie Orth, Selina Jawahir, Annastasia Gross, Xiong Wang, Dave Boxrud, Kirk Smith, Jisun Haan

**Affiliations:** 1 Minnesota Department of Health 11055, Saint Paul, Minnesota, USA; 2 Scrippes Research 11055, La Jolla, California, USA; 3 Enteric Diseases Laboratory Branch, Centers for Disease Control and Prevention 1242, Atlanta, Georgia, USA; Mayo Clinic, Rochester, Minnesota, USA

**Keywords:** human campylobacteriosis, antimicrobial resistance, virulence factors, MDR, *Campylobacter*

## Abstract

*Campylobacter jejuni* is a leading cause of foodborne gastroenteritis globally and is classified by the Centers for Disease Control and Prevention as a serious public health threat due to increasing resistance to fluoroquinolones and macrolides. This study used whole-genome sequencing to characterize the virulence and antimicrobial resistance profiles of 2,783 clinical *C. jejuni* isolates (98.4% stool, 1.2% unknown source, 0.4% blood, and one from rectal swab) collected from Minnesota residents from 2018 through 2021. More than 90% of the isolates had genes related to stress defense (*rpoN* and *htrB*), cytolethal distending toxin (*cdtA*, *cdtB*, and *cdtC*), and cell adhesion and invasion (*ciaB*, *cadF*, and *flaC*). A diverse array of antimicrobial resistance genes were detected, with beta-lactam resistance genes having a particularly high prevalence. Five *gyrA* point mutations associated with quinolone resistance were present in 29% of isolates, with the T861 substitution being the most prevalent. Phenotypically, tetracycline resistance was most common (52.3%), followed by quinolone resistance (30.7%), while resistance to other antimicrobial classes—macrolide (2.4%) and aminoglycoside (1.8%)—was uncommon. To evaluate the correlation between genotypic and phenotypic antimicrobial resistance profiles, the antimicrobial susceptibility testing results from a subset of isolates were compared with genotypic resistance profiles. The results showed a strong overall correlation, particularly for tetracycline and quinolones, though 24 discrepancies were detected. In the majority of discrepancies (*n* = 21), genomic antimicrobial resistance markers were absent in isolates that were phenotypically resistant, suggesting possible unknown resistance mechanisms or limitations in current sequencing analysis methods. The remaining three discrepancies occurred in isolates that had the *tet*(O) resistance gene but were susceptible to tetracycline phenotypically. These findings highlight the value of whole-genome sequencing in improving antimicrobial resistance surveillance and understanding virulence-associated factors in *C. jejuni*, supporting its integration into routine monitoring practices to better manage and understand antimicrobial resistance in foodborne pathogens.

**IMPORTANCE:**

This study characterizes the virulence and antimicrobial resistance profiles of *Campylobacter* isolates using whole-genome sequencing (WGS) data generated through routine foodborne disease surveillance. A subset of isolates was also tested for phenotypic antimicrobial susceptibility testing, allowing us to evaluate resistance patterns and assess genotype-phenotype concordance. This comparison provides important evidence regarding the reliability and limitations of using WGS-detected resistance determinants to predict antimicrobial susceptibility. The 5 years of accumulated surveillance data provide a comprehensive, population-level understanding of circulating *Campylobacter* strains in Minnesota, USA, and establish an important regional baseline for monitoring changes over time. These findings can be compared with national surveillance data to identify geographic similarities and differences and provide a valuable regional context for public health decision-making and antimicrobial stewardship.

## INTRODUCTION

*Campylobacter jejuni* is one of the leading causes of bacterial gastroenteritis in humans globally (1). In healthy individuals, campylobacteriosis is typically self-limiting (2). However, severe infections can occur, especially in at-risk populations, such as children, the elderly, and the immunocompromised (3). In such cases, antibiotic treatment with macrolides or fluoroquinolones is often necessary (3). There is a growing concern over the increasing resistance of *C. jejuni* to antibiotics worldwide (4, 5), which poses significant public health risks. The National Antimicrobial Resistance Monitoring System (NARMS) 2019 Integrated Report indicated that the percentage of fluoroquinolone-resistant *C. jejuni* in human isolates increased from 29% in 2018 to 34% in 2019 (6). Similar increases in resistance were observed in chicken retail samples and cecal contents (6), reducing the effectiveness of fluoroquinolone treatment for campylobacteriosis.

The natural reservoirs of *C. jejuni* include a wide range of wild and domesticated animals, including wild birds, cattle, sheep, and poultry. Consumption of undercooked or raw poultry products is considered the major source of *C. jejuni* in sporadic campylobacteriosis in humans (7), while consumption of unpasteurized milk or contaminated water is often linked to outbreaks (8).

Pulsed-field gel electrophoresis (PFGE) was considered a gold-standard method for foodborne bacterial pathogen subtyping performed by public health laboratories (9). In 2019, the PulseNet National Laboratory Network transitioned from PFGE to whole-genome sequencing (WGS) for the detection and investigation of nationwide foodborne disease outbreaks in the United States, improving the discrimination and precision of investigations (9). WGS can generate high-resolution genetic information with relatively quick turnaround time at a reasonable cost. The sequencing data generated by WGS allow comprehensive phylogenetic analysis of a set of bacterial strains, as well as screening of molecular markers of virulence and antimicrobial resistance (AMR).

The Minnesota Department of Health, Public Health Laboratory (MDH-PHL) has performed routine WGS of all available *Campylobacter* isolates since 2018 to aid in outbreak detection and investigation. However, extensive genomic characterization of these isolates has not been conducted. In this study, we used WGS to provide comprehensive genotypic AMR and virulence-associated factor profiles. Phenotypic resistance data from a subset of clinical isolates were compared to the genotypic AMR profiles to evaluate the accuracy of WGS in predicting phenotypic antimicrobial resistance in *C. jejuni*.

## MATERIALS AND METHODS

### Bacterial isolate collection

*Campylobacter* is a reportable pathogen according to Minnesota Reportable Disease Rule, 4605.7040. As a part of Foodborne Diseases Active Surveillance Network (FoodNet) activities, the MDH conducts active surveillance to ascertain 100% of laboratory-confirmed *Campylobacter* infections in Minnesota residents. Specimens that test *Campylobacter*-positive by culture-independent diagnostic testing and *Campylobacter* isolates must be submitted to the MDH-PHL for all cases of campylobacteriosis; the MDH-PHL attempts to isolate *Campylobacter* from all submitted positive clinical specimens and identifies all *Campylobacter* isolates to species. The *C. jejuni* isolates included in this study comprised all 3,628 clinical *C. jejuni* isolates collected from Minnesota residents from 2018 through 2021.

### Isolation and identification of *Campylobacter jejuni*

When a clinical laboratory submitted a positive clinical specimen to the MDH-PHL, *Campylobacter* sp. isolation was attempted from stool samples or rectal swabs submitted in Cary-Blair or other enteric transport media using a filtration method at the MDH-PHL. A cellulose acetate filter (0.65 µm) was placed on two *Campylobacter* blood agar (CBA) plates prepared in-house without selective supplements, and 6–8 drops of the stool sample were directly placed on the filter. After incubating the plate for 45 min to 1 h at 36°C, the filter was removed. One plate was incubated at 42°C for 24–72 h under microaerophilic conditions (87.4% N_2_, 7.6% CO_2_, and 5% O_2_), and the other was incubated at 36°C for 48 h under increased hydrogen microaerophilic conditions (81.3% N_2_, 8.6% CO_2_, 7.1% H_2_, and 3% O_2_). The clinical specimen was also directly plated to a cefoperazone, vancomycin, and amphotericin B agar (CVA) plate (Hardy Diagnostics, USA) for isolation and incubated at 42°C for 24–72 h under microaerophilic conditions.

An oxidase test was performed on two to three *Campylobacter*-like colonies from CBA and CVA plates. Colonies with a positive oxidase test were sub-cultured to CBA and incubated for 24 h at 42°C under microaerophilic conditions. Gram’s stain and matrix-assisted laser desorption/ionization-time of flight mass spectrometry (MALDI-TOF MS) identification (Bruker, USA) were performed to determine the species of the *Campylobacter* isolate.

Gram’s stain, oxidase, and MALDI-TOF MS identification were performed to confirm the species when *C. jejuni* isolates were submitted instead of clinical specimens.

### Whole-genome sequencing and genome assembly

*Campylobacter* genomic DNA was extracted on QIAcube Classic using the QIAamp DNA Mini QIAcube kit (Qiagen, Germany) or MagNA Pure LC (Roche, Switzerland) using the Pure LC Total Nucleic Acid (TNA) Isolate kit (MagNA Pure) following the manufacturer’s instructions. DNA concentration was measured using the Quant-iT dsDNA HS Assay kit (Invitrogen, USA). Libraries were prepared using the Nextera XT index kit (Illumina, USA) following the manufacturer’s instructions. Sequencing was performed on the Illumina Miseq platform with 250-bp-paired-end reads using V2 chemistries. Raw reads were trimmed and quality-filtered using Trimmomatic PE (10). High-quality reads were *de novo-*assembled by Skesa (ver. 2.4.0) using default parameters (11). Assembly metrics were calculated using QUAST (ver. 5.0.2) with *C. jejuni* NCTC 11168 as a reference genome (BioSample: SAMEA1705929; SRA: ERS000062) (12). BUSCO (ver. 5.2.2) was performed to assess genome assembly completeness (13). Lastly, the average genome coverage was calculated using Samtools (ver. 1.15) (14). Assembled genomes were excluded from further analysis using the following exclusion criteria: (i) N50 <10,000; (ii) total length of the assembled genome >2,200,000 bps; (iii) genome completeness by BUSCO <95%; and (iv) average genome coverage <20×. The final data set used for the downstream analyses consisted of 2,783 genomes.

### WGS analysis: AMR prediction and virulence-associated factor screening

The presence of acquired resistance genes and chromosomal point mutations were screened using ResFinder (ver. 4.0) and BLASTn (ver. 2.9.0), respectively (15, 16). The minimum identity threshold for matching was set to 90% with 60% minimum coverage. Virulence-associated factor screening was performed using ABRicate (ver. 1.0.1) against the Virulence Factor Database (VFDB) using the default parameters (17, 18). A virulence-associated factor was considered present if the minimum proportion of the gene covered (coverage) was greater than 90%. Specifically, the distribution of 17 virulence genes associated with cytolethal distending toxin (CDT)-producing genes (*cdtA*, *cdtB*, and *cdtC*), cell invasion and adhesion (*ciaB*, *ciaC*, *flaC*, *cadF*, and *jlpA*), motility (*flaA* and *flaB*), stress response (*rpoN* and *htrB*), and Type IV secretion system (*virB8*, *virB9*, *virB10*, *virB11*, and *virD4*) was investigated.

### Antimicrobial susceptibility testing

A subset (*n* = 547) of *C. jejuni* isolates from the MDH-PHL was selected to measure the minimum inhibitory concentration (MIC) using broth microdilution. These clinical isolates were selected randomly, with every 20th isolate submitted for antimicrobial susceptibility testing (AST) as part of the NARMS surveillance. Susceptibility to nine antibiotics was measured: azithromycin, ciprofloxacin, erythromycin, tetracycline, florfenicol, nalidixic acid, telithromycin, clindamycin, and gentamicin. Briefly, 3–5 isolated colonies were suspended in 5 mL of Mueller Hinton broth with TES (Thermo Fisher Scientific, USA). The suspension was adjusted to 0.5 McFarland turbidity, and 100 µL of the suspension was diluted in 11 mL Mueller Hinton broth with lysed horse blood (Thermo Fisher Scientific, USA). SensiTitre *Campylobacter* CMVCAMPY panels, a surveillance/RUO (Research Use Only) panel, (Thermo Fisher Scientific, USA) were inoculated with 100 µL of the inoculum using TREK SensiTitre AIM Automated Inoculation Delivery System (TREK Diagnostic Systems, USA) with *C. jejuni* ATCC 33560 as a quality control strain. Panels were incubated at 37°C for 48 h under microaerophilic conditions. Panels were manually read using SensiTitre Vizion Digital MIC viewing system (TREK Diagnostic Systems, USA), and MICs were interpreted as susceptible or resistant using epidemiological cutoff values (ECVs) established by the European Committee on Antimicrobial Susceptibility Testing. The breakpoints used for each antimicrobial can be found at the CDC NARMS website (https://www.cdc.gov/narms/about/antibiotics-tested.html) (19). After data collection, trends in the prevalence of antimicrobial resistance were analyzed for three antimicrobial agents—ciprofloxacin, tetracycline, and nalidixic acid. Other agents were excluded from the analysis due to the low prevalence of resistance. The original *P* values from odds ratios (ORs) were adjusted for multiple comparisons by year using the Benjamini-Hochberg method (20).

### Phenotypic and genotypic AMR profile comparison

The Cohen’s Kappa statistic was calculated to assess the level of concordance between the resistance phenotype obtained from the broth microdilution method and the genotype obtained using ResFinder in RStudio (ver. 4.1.2) (21). The resistance genotype was defined as the presence of one or more resistance genes and/or point mutations for each antimicrobial tested using the broth microdilution method.

## RESULTS

### Genome characteristics

A total of 3,628 *C. jejuni* isolates from human clinical cases collected in Minnesota from 2018 through 2021 were initially obtained for the study. Following the application of filtering criteria, including genome completeness, average genome coverage, total genome length, and N50 of the assembled genome, 2,783 isolates were included in the final analysis (Table S1). Of these, 912 isolates were collected in 2018, 739 in 2019, 484 in 2020, and 648 in 2021. The majority of the isolates (*n* = 2,739) were from stool or rectal swabs, with 10 from blood and 34 from other specimen types.

The average GC content of the compiled genomes was 30.4% (range: 29.9%–31.4%). The average length of the assembled genomes was 1,677,537 bps (range: 1,533,180–1,961,390 bps) with an average genome completeness of 98.8% (range: 95.1%–99.8%). The average genome coverage was 57.8× (range: 20.1–558.01×).

### Virulence factor screening

Two virulence genes, *cadF* and *flaC*, which are involved in cell adhesion and invasion, were present in all 2,783 isolates (Table 1). The CDT-producing genes (*cdtA*, *cdtB*, and *cdtC*), stress defense genes (*rpoN* and *htrB*), and two cell adhesion and invasion genes (*ciaC* and *jlpA*) were present in more than 95% of the isolates, while *ciaB* was found in 75% of the isolates. Approximately a quarter of isolates carried *flaA* and *flaB*, while less than 6% of the isolates carried the Type IV secretion system virulence genes.

**TABLE 1:** Prevalence of virulence-associated genes in 2,783 clinical *C. jejuini* isolates from Minnesota residents, 2018–2021

Virulence factor	Gene	Number of isolates (%)
Cytolethal distending toxin	*cdtA*	2,712 (97.4)
	*cdtB*	2,739 (98.4)
	*cdtC*	2,766 (99.4)
Cell invasion and adhesion	*ciaB*	2,093 (75.2)
	*ciaC*	2,782 (99.9)
	*flaC*	2,783 (100)
	*cadF*	2,783 (100)
	*jlpA*	2,688 (96.6)
Motility	*flaA*	688 (24.7)
	*flaB*	792 (28.5)
Stress-defense	*rpoN*	2,780 (99.9)
	*htrB*	2,779 (99.9)
Type IV secretion system	*virB8*	155 (5.6)
	*virB9*	155 (5.6)
	*virB10*	156 (5.6)
	*virB11*	152 (5.5)
	*virD4*	151 (5.4)

### Genotypic antimicrobial resistance

ResFinder was run on 2,783 *C. jejuni* isolates to identify antimicrobial resistance genes and point mutations (Tables S2 and S3). Twenty-five acquired antimicrobial resistance genes and nine chromosomal point mutations were identified. The distribution of acquired antimicrobial resistance genes and chromosomal point mutations is presented in Tables 2 and 3, respectively. The majority of the isolates (*n* = 2,571, 92.4%) had at least one acquired resistance gene or point mutation associated with antimicrobial resistance, while 212 (7.6%) were genotypically pan-susceptible. Among the isolates with antimicrobial resistance determinants, 2,520 (90.5%) had at least one acquired resistance gene, and 837 (29.7%) had at least one point mutation associated with antimicrobial resistance.

**TABLE 2:** Distribution of antimicrobial resistance genes in clinical *C. jejuni* isolates (*n* = 2,520) from Minnesota residents, 2018–2021

Drug class	Antimicrobial resistance gene	Number of isolates with gene (%) (*n* = 2,520)
Macrolides	*ermB*	1 (0.04)
Lincosamides	*lnuC*	1 (0.04)
Tetracyclines	*tet*(O)	1,331 (52.8)
	*tet*(O/32/O)	81 (3.2)
	*tet*(O/W/32/O)	2 (0.08)
Beta-lactams	*bla*_OXA61_	1,617 (64.2)
	*bla*_OXA184_	96 (3.8)
	*bla*_OXA185_	14 (0.6)
	*bla*_OXA193_	1,533 (60.8)
	*bla*_OXA446_	2 (0.08)
	*bla*_OXA447_	31 (1.2)
	*bla*_OXA448_	129 (5.1)
	*bla*_OXA449_	45 (1.8)
	*bla*_OXA450_	1,472 (58.4)
	*bla*_OXA451_	1,470 (58.4)
	*bla*_OXA453_	1,481 (58.8)
	*bla*_OXA460_	129 (5.1)
	*bla*_OXA461_	145 (5.8)
	*bla*_OXA465_	41 (1.6)
	*bla*_OXA466_	34 (1.3)
	*bla*_OXA489_	1,474 (58.5)
Aminoglycosides	*aph*3′-III	278 (11)
	*aph(2*″)*-Ih*	13 (0.5)
	*aph(2*″)*-If*	7 (0.3)
	ant(6′)-Ia	26 (1)

**TABLE 3:** Distribution of point mutations in clinical *C. jejuni* isolates from Minnesota residents, 2018–2021

Gene	Point mutation(s)	Number of isolates with mutation (%) (*n* = 837)
*gyrA* (*n* = 821)	T86I	811 (96.8)
	T86A	1 (0.1)
	T86V	1 (0.1)
	T86I + D90N	6 (0.7)
	T86I + P104S	2 (0.2)
23S rRNA (*n* = 53)	S2075AG	41 (5.0)
	S2074AT	12 (1.4)
*rpsL* (*n* = 4)	K43R	2 (0.2)
	K88R	2 (0.2)

Among the 25 acquired resistance genes, *bla*_OXA-61_ (*n* = 1,617; 64.2%) was, by far, the most frequently identified. Multiple *bla*_OXA_ genes, which are involved in encoding β-lactamase, were identified in individual isolates. In addition to *bla*_OXA-61,_ the genes *bla*_OXA-489_, *bla*_OXA-453_, *bla*_OXA-451_, *bla*_OXA-450_, and *bla*_OXA-193_ were also commonly detected (Table 2). The second-most frequently observed resistance gene was *tet*(O) (*n* = 1,331, 52.8%). In addition to *tet*(O), the mosaic tetracycline genes *tet*(O/32/O) (*n* = 81, 3.2%) and *tet*(O/W/32/O) (*n* = 2, 0.08%) were also identified, though at a lower frequency. Four resistance genes associated with aminoglycosides were identified, with *aph(3')-III* (*n* = 278; 11%) being the most frequently observed aminoglycoside resistance gene among the isolates. Notably, *aph(2'')-If*, which has been reported to confer resistance to kanamycin and gentamicin in *C. jejuni* (22), was detected in seven isolates.

The most common point mutations were *gyrA* point mutations associated with fluoroquinolone resistance (*n* = 821, 98.1%) (Table 3). The T86I (*n* = 811, 96.8%) mutation was the most common *gyrA* point mutation, followed by double-point mutations of T86I + D90N (*n* = 6, 0.7%), T86I + P104S (*n* = 2, 0.2%), T86A (*n* = 1, 0.1%), and T86V (*n* = 1, 0.1%). Notably, the less common *gyrA* point mutations, D90N and P104S, were detected together with T86I (*n* = 6, *n* = 2, respectively). A total of 53 (6.3%) *C*. *jejuni* isolates had point mutations in the 23S rRNA that confer resistance to azithromycin, erythromycin, clindamycin, and telithromycin. Four isolates had a point mutation in the *rpsL* gene, which confers resistance to streptomycin.

### Phenotypic antimicrobial resistance

**Fig 1:**
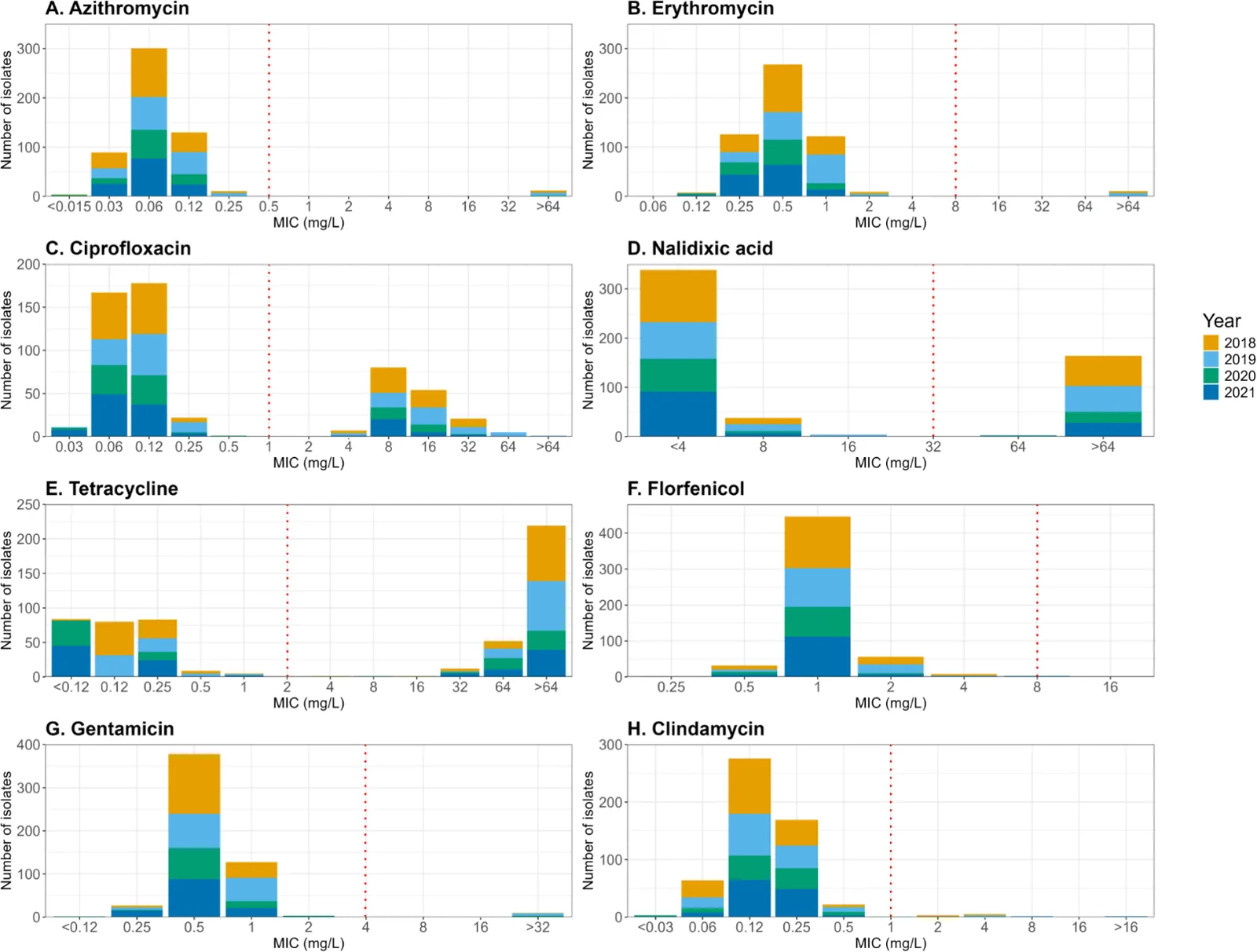
Distribution of MIC values for eight antibiotics (**A–H**) from a subset of clinical *C. jejuni* isolates from Minnesota residents, 2018–2021. The resistance breakpoint concentration is denoted by a red dotted line.

The MICs of a subset of clinical *C. jejuni* isolates (*n* = 547) are presented in Fig. 1. These isolates were randomly selected to test MICs as part of NARMS surveillance. A total of 218 (40%) of these were pan-susceptible to all antibiotics tested. The most frequently observed resistance phenotype was tetracycline (*n* = 286, 52.3%), followed by quinolones (*n* = 168, 30.7%), macrolides (*n* = 13, 2.4%), lincosamides (*n* = 13, 2.4%), aminoglycosides (*n* = 10, 1.8%), and phenicols (*n* = 4, 0.7%) (Table S5). One isolate was resistant to ciprofloxacin but susceptible to nalidixic acid (Table S4).

**Fig 2:**
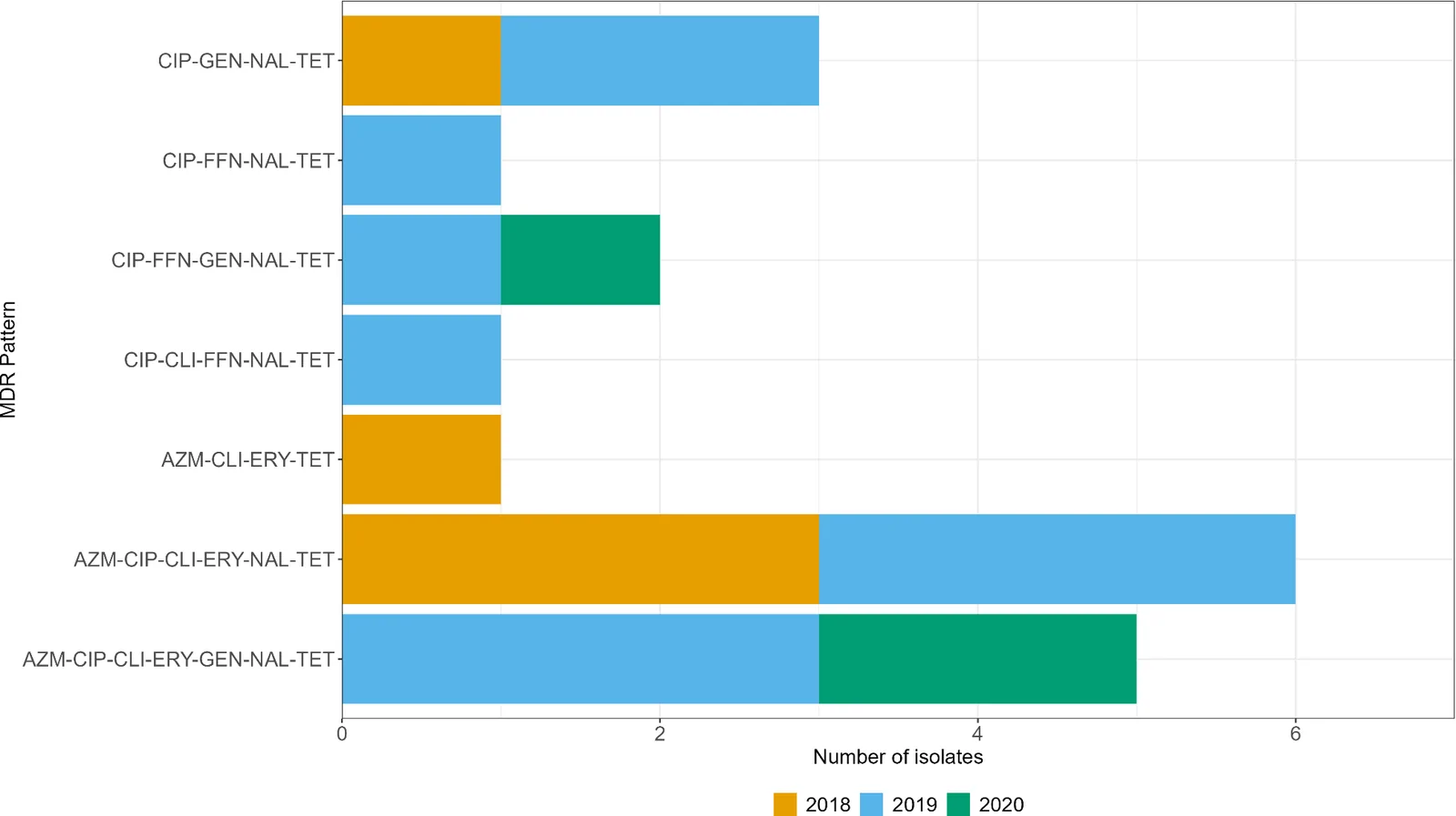
Distribution of multidrug resistance patterns among a subset of clinical *C. jejuni* isolates from Minnesota residents, 2018–2021. CIP, ciprofloxacin; GEN, gentamicin; NAL, nalidixic acid; TET, tetracycline; FFN, florfenicol; CLI, clindamycin; AZM, azithromycin; ERY, erythromycin.

Nineteen isolates (3.5%) were multidrug-resistant (MDR) (resistant to three or more antibiotic classes) (Fig. 2). The most common MDR pattern was AZM-CIP-CLI-ERY-NAL-TET (*n* = 6). Five MDR isolates were resistant to five classes of antibiotics (macrolides, quinolones, aminoglycosides, lincosamides, and tetracyclines).

**Fig 3:**
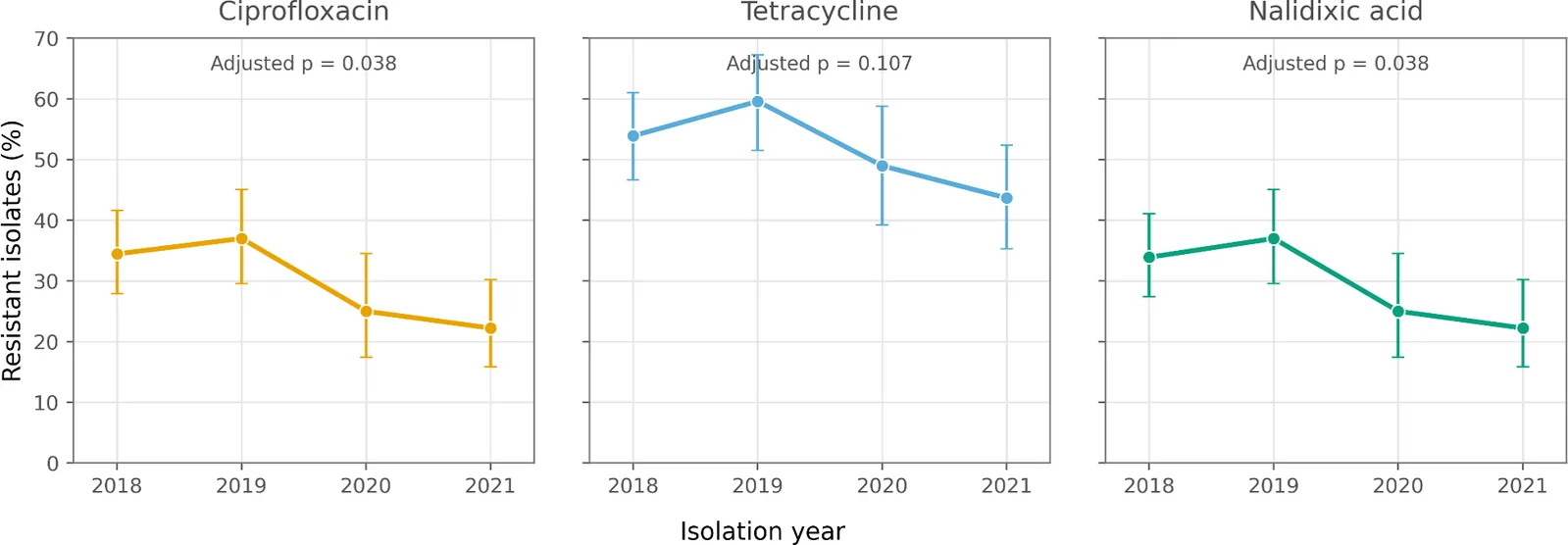
Prevalence of isolates resistant to ciprofloxacin, tetracycline, and nalidixic acid, 2018–2021. Resistance to ciprofloxacin and nalidixic acid showed significant decreasing trends over the study period (adjusted *P* = 0.038 for each).

Resistance to the quinolones ciprofloxacin and nalidixic acid decreased significantly with isolation year. The ORs per year were 0.80 (95% CI, 0.68–0.94; adjusted *P* = 0.038) for ciprofloxacin and 0.81 (95% CI, 0.69–0.95; adjusted *P* = 0.038) for nalidixic acid (Fig. 3). For tetracycline, the OR per year was 0.86 (95% CI, 0.74–0.99; adjusted *P* = 0.107), which was not statistically significant after adjustment for multiple comparisons. The largest decreases for all three antimicrobial agents were observed between 2019 and 2020, although none of the individual year-to-year comparisons was statistically significant. No significant linear trends were identified for other antimicrobial agents because the resistance prevalence was low, ranging from 0% to 4.8%.

### Comparison between phenotypic and genotypic antimicrobial resistance profiles

Overall, high concordance between phenotypic resistance and genotypic resistance was observed across all antibiotic classes tested, with the average Cohen’s Kappa statistic of 0.89 (Table 4). A total of 24 mismatches were observed, and mismatches occurred across all antibiotic classes. A majority (*n* = 21) of the mismatches occurred because isolates were phenotypically resistant to the antibiotics but lacked resistance genes or point mutations that conferred the phenotypic resistance. This type of mismatch was most frequently observed with tetracycline (*n* = 11), followed by quinolones (*n* = 5), lincosamides (*n* = 3), aminoglycosides (*n* = 3), and macrolides (*n* = 2). The other three mismatches occurred in the isolates that carried *tet*(O) but were phenotypically susceptible to tetracycline, with their MICs against tetracycline below 1 µg/mL. More than one mismatch was observed in three isolates. For instance, the isolate SRR7750849 was phenotypically resistant to tetracycline and ciprofloxacin yet lacked the *gyrA* point mutation and tetracycline resistance gene (SRR7750849 in Tables S3 to S5).

**TABLE 4:** Correlation of the resistance phenotype and predicted genotype of clinical *C. jejuni* isolates

Drug class	No. of isolates resistant (%) based on phenotypic testing (*n* = 547)	Resistance gene/point mutation	Cohen’s Kappa statistics (standard error)
Quinolones	Ciprofloxacin (168, 30.7%)	*gyrA* T86I (*n* = 163)	0.98 (0.009)
	Nalidixic acid (167, 30.5%)	*gyrA* T86I (*n* = 163)	0.98 (0.008)
Tetracycline	Tetracycline (286, 52.3%)	*tet*(O) (*n* = 261) *tet*(O/32/O) (*n* = 19) *tet*(O/W/32/O) (*n* = 1)	0.96 (0.01)
Macrolides	Azithromycin (13, 2.4%)	23S rRNA A2075G (*n* = 10) 23S rRNA A2074T (*n* = 1)	0.91 (0.06)
	Erythromycin (13, 2.4%)	23S rRNA A2075G (*n* = 10) 23S rRNA A2074T (*n* = 1)	0.91 (0.06)
Aminoglycosides	Gentamicin (10, 1.8%)	*aph(2*″)*-If* (*n* = 2)	0.66 (0.14)
Lincosamides	Clindamycin (13, 2.4%)	23S rRNA A2075G (*n* = 10)	0.87 (0.07)

## DISCUSSION

In our study, more than 97% of clinical isolates from Minnesota patients had CDT-producing genes, *cdtA*, *cdtB*, and *cdtC*. The CDT has been shown to induce DNA damage, which ultimately results in cell death and compromises the integrity of the intestinal epithelium (23, 24). However, a study by Abuoun et al. recovered CDT-negative *C. jejuni* from clinical stool samples, albeit infrequently, indicating that additional mechanisms besides CDT can cause clinical symptoms of campylobacteriosis in humans (25). Nonetheless, the prevalence of CDT-producing genes was also high in other studies, suggesting that these genes are widely distributed among *C. jejuni* isolates (26–29).

Only a quarter of the clinical isolates had *flaA* and *flaB*, two flagellin genes responsible for motility in *C. jejuni*. Both functional *flaA* and *flaB* genes are required for the maximum motility in *C. jejuni* (30). Previous studies have shown that non-motile *C. jejuni* was able to colonize chickens, however, at a reduced ability or for a short term, indicating that motility is an important virulence factor as it allows *C. jejuni* to migrate and move within the thick mucosal lining of the intestinal tract (31–33). Although flaA and/or flaB were not detected in 2/3 isolates, the majority of isolates (>98%) were shown to possess other virulence genes (i.e, flaC, D, G, and flgA-K, P, R, S, and M), which indicates that those strains are able to attach the human epithelial cells and trigger cell invasion without the major structure filament proteins to complete the tails. This suggests that some of the strains are highly distinct and well-adapted clinical lineage of *C. jejuni*. A similar prevalence of *flaA/B* has been reported by Panzenhagen et al., which looked at the distribution of virulence factors in *C. jejuni* sequences deposited in publicly available databases (29). Possible explanations for such a low prevalence provided by Panzenhagen et al. were homologous recombination and intragenic recombination (29). Recombination, which occurs frequently in *C. jejuni*, is thought to be the major source of genetic diversity in *C. jejuni* (34).

The *virB11* virulence-associated gene is located on the pVir plasmid. In our study, *virB11* and other type IV secretion system genes were found only in 5% of the isolates. According to a study by Wysok et al., the absence of *virB11* in *C. jejuni* led to a slight reduction in HeLa cell invasion, while the absence of the *iam* gene, an invasion-associated marker, significantly reduced cell invasion (35). The prevalence of the *iam* gene was higher than that of *virB11* in the study by Wysok et al. as well. Coupled with the reported low prevalence of *virB11* in *C. jejuni* in chickens and humans (36, 37), the *virB11* gene may not be a critical virulence-associated factor for campylobacteriosis in humans (36, 38). Nevertheless, the presence or absence of *virB11* alone should not be interpreted as evidence of an isolate’s virulence or pathogenic potential, and its contribution to *Campylobacter* pathogenesis *in vivo* remains uncertain.

To perform genome-based prediction of AMR, assembled genomes were screened through a curated ResFinder database. In our study, 29% of clinical isolates carried a *gyrA* point mutation, which confers resistance to quinolones. This level of resistance was similar to the prevalence of quinolone-resistant *C. jejuni* in humans and retail chickens reported by the NARMS (6). Five different *gyrA* point mutations were detected. Of these, T86I, which is associated with a high level of fluoroquinolone resistance in *C. jejuni*, was the most frequently observed mutation, consistent with previously reported observations (39). We also detected D90N and P104S mutations, which have previously been detected in clinical isolates (40–42). Interestingly, these mutations were detected together with the T86I mutation. While the D90N mutation is associated with an intermediate level of fluoroquinolone resistance (43), the P104S mutation on its own has been detected in phenotypically sensitive isolates and requires an additional mutation, T86I, to be phenotypically resistant (44). It has been suggested that stepwise accumulation of the *gyrA* point mutation does not lead to an increased level of quinolone resistance (45). Therefore, it is unclear how the acquisition of additional *gyrA* point mutations, P104S and D90N, might affect the levels of resistance to quinolones.

A high percentage of isolates carried beta-lactam resistance genes including several blaOXA alleles, *bla*_OXA-61_, *bla*_OXA-193_, *bla*_OXA-450_, *bla*_OXA-451_, *bla*_OXA-453_, and *bla*_OXA-489_. Since *Campylobacter* is known to be intrinsically resistant to beta-lactam antimicrobials and narrow-spectrum cephalosporins (3), ampicillin or any other beta-lactam antibiotics have limited application in the treatment of campylobacteriosis (46), and the screening of beta-lactam resistance genes in *C. jejuni* is rarely conducted. However, WGS is a useful tool for understanding resistance modulation mechanisms in addition to identifying novel resistance genes (47–49). For example, it has been shown that the single-nucleotide mutation upstream of *bla*OXA_61_, which modulates the expression of *bla*OXA_61_, might be responsible for ampicillin resistance in *C. jejuni* rather than the presence of the gene itself (50) as *bla*OXA_61_-carrying *C. jejuni* isolated from poultry was found to be susceptible to ampicillin (51, 52).

More than 50% of the isolates in the study had a tetracycline resistance marker, consistent with observations reported by other studies (47, 53–55). The *tet*(O) was the most frequently detected tetracycline marker; however, two mosaic *tet*(O) genes were detected at a lower frequency. The mosaic *tet* genes are proven to be functional and confer resistance to tetracycline (56). Surveillance of mosaic *tet* genes has been done primarily using PCR-based assays. However, this approach requires knowledge of the target gene sequences, likely to result in an underestimation of *tet* resistance in *C. jejuni*. Implementing WGS to conduct AMR surveillance in foodborne pathogens can improve our understanding of the prevalence and diversity of mosaic tetracycline genes (57).

AST was performed on a subset of the isolates to compare phenotypic and genotypic antimicrobial resistance patterns. Similar to the genotype prediction results based on WGS, 30% of the isolates were resistant to quinolone antibiotics—ciprofloxacin and nalidixic acid—based on phenotypic testing, and a high prevalence of tetracycline resistance (52%) was observed. A significant decrease in ciprofloxacin, nalidixic acid, and tetracycline resistance over the study period was also observed, although tetracycline resistance showed a decreasing trend, but it was not statistically significant. These findings may reflect changes in the epidemiology and circulating populations of *C. jejuni* in Minnesota. The largest decline was found between 2019 and 2020 (10%–12% decrease), suggesting any exposure changes associated with the COVID-19 pandemic. Quinolone resistance is often more associated with international travel-related infections than domestically acquired infections (58). Therefore, restrained habits and decreased numbers of international travel during the pandemic may have contributed to the decrease in quinolone resistance. The CDC COVID-19 AMR report also supports our data, describing a 10% decrease from 2019 to 2020 in *Campylobacter* isolates with decreased susceptibility to ciprofloxacin or azithromycin (59).

When resistance phenotype profiles were compared to the genotypic prediction, we observed only 24 discrepancies out of 547 isolates compared. The majority of the observed discrepancies occurred when isolates were phenotypically resistant but lacked the known genetic markers. Notably, three isolates showed multiple discrepancies. For example, one isolate was phenotypically resistant to macrolides, clindamycin, and quinolones but lacked known point mutations in the 23s rRNA gene and *gyrA* gene. In addition, four isolates were phenotypically resistant to ciprofloxacin, but no point mutation was detected in *gyrA* that could explain the observed phenotype. This result can be attributed to sequencing artifacts or unknown resistance mechanisms in *C. jejuni*. The cmeABC efflux pump plays an important role in antimicrobial resistance in *C. jejuni* by removing various antimicrobial agents (60). Previous studies have shown that increased expression of *cmeA*, a subunit of the cmeABC operon, led to increased resistance to tetracycline and ciprofloxacin (53, 61). Differential expression of *cmeA* was also observed between ciprofloxacin-susceptible and resistant *C. jejuni* (53), suggesting additional molecular mechanisms conferring resistance to fluoroquinolone antibiotics besides *gyrA* point mutations. Further investigation is needed to assess *cmeA* expression levels and determine their correlation with phenotypic quinolone resistance and the presence of known genetic resistance determinants. Notably, three isolates carried the *tet*(O) gene but were phenotypically susceptible, with their MICs for tetracycline ≤0.25 µg/mL. The detection of the *tet*(O) gene in clinical and poultry *C. jejuni* isolates has shown that *tet*(O) can be present on a plasmid rather than located on the chromosome (62, 63). It is plausible that while these three isolates carried the *tet*(O) genes, they were located on a plasmid and were not expressed, resulting in phenotypic susceptibility to tetracycline.

Overall, our results show a high level of concordance between genotypic AMR prediction and phenotypic resistance, consistent with findings from several other studies (53, 64–66). NARMS now employs WGS data to predict AMR in enteric bacteria, including *Salmonella* and *Campylobacter*. Their findings demonstrate that WGS-based AMR prediction correlates well with phenotype data (MICs) for most antimicrobial drugs, supporting the use of WGS as a rapid and reliable method for AMR screening (67). However, standardization of WGS methods, use of bioinformatics tools, and AMR reference databases may be warranted to standardize experimental procedures across different laboratories to achieve comprehensive and accurate results.

In conclusion, our study identified a high prevalence of key virulence-associated factors, such as CDT-producing genes, and highlighted the genetic diversity of resistance genes, particularly those associated with beta-lactam resistance. A high correlation between genotypic and phenotypic AMR profiles was detected for tetracyclines and quinolones, although discrepancies detected indicate potential gaps in our understanding of resistance mechanisms or limitations with current sequencing technologies. These findings highlight the utility of WGS in enhancing our understanding of AMR and virulence in *C. jejuni*. Importantly, this study provides a regional baseline for the distribution of antimicrobial resistance and genotype-phenotype concordance in the *Campylobacter jejuni* population collected in Minnesota over 4 years. Regional surveillance plays an essential role in identifying temporal trends, detecting emerging resistance patterns, and generating population-level data that can be compared with the national surveillance database. Overall, incorporating WGS into routine, population-based AMR monitoring could improve our ability to detect and manage antimicrobial resistance in foodborne pathogens.

## Data Availability

The data sets supporting the conclusions of this study have been deposited in the National Center for Biotechnology Information (NCBI). Individual accession numbers are provided in Table S1.

## SUPPLEMENTAL MATERIAL

Tables S1 to S5 jcm.00514-26-s0001.xlsx Includes raw data of sequence quality, metrics, sequencing results, and AMR results.

ASM does not own the copyrights to Supplemental Material that may be linked to, or accessed through, an article. The authors have granted ASM a non-exclusive, world-wide license to publish the Supplemental Material files. Please contact the corresponding author directly for reuse.

## References

[1] Kaakoush NO, Castaño-Rodríguez N, Mitchell HM, Man SM. 2015. Global epidemiology of *Campylobacter* infection. Clin Microbiol Rev 28:687–720. doi:10.1128/CMR.00006-15 PMC4462680 26062576

[2] Galanis E. 2007. *Campylobacter* and bacterial gastroenteritis. Can Med Assoc J 177:570. doi:10.1503/cmaj.070660 PMC1963361 17846438

[3] Wieczorek K, Osek J. 2013. Antimicrobial resistance mechanisms among *Campylobacter*. Biomed Res Int 2013:340605. doi:10.1155/2013/340605 PMC3707206 23865047

[4] Lopez-Chavarrias V, Ugarte-Ruiz M, Barcena C, Olarra A, Garcia M, Saez JL, de Frutos C, Serrano T, Perez I, Moreno MA, Dominguez L, Alvarez J. 2021. Monitoring of antimicrobial resistance to aminoglycosides and macrolides in *Campylobacter coli* and *Campylobacter jejuni* from healthy livestock in Spain (2002–2018). Front Microbiol 12:689262. doi:10.3389/fmicb.2021.689262 PMC8283307 34276619

[5] European Food Safety Authority, European Centre for Disease Prevention and Control. 2019. The European Union summary report on antimicrobial resistance in zoonotic and indicator bacteria from humans, animals and food in 2017. EFSA J 17:e05598. doi:10.2903/j.efsa.2019.5598 PMC7009238 32626224

[6] FDA. 2024. NARMS now - integrated data. NARMS Integr Data. Available from: https://www.fda.gov/animal-veterinary/national-antimicrobial-resistance-monitoring-system/narms-now-integrated-data

[7] Friedman CR, Hoekstra RM, Samuel M, Marcus R, Bender J, Shiferaw B, Reddy S, Ahuja SD, Helfrick DL, Hardnett F, Carter M, Anderson B, Tauxe RV, Emerging Infections Program FoodNet Working Group. 2004. Risk factors for sporadic *Campylobacter* infection in the United States: a case-control study in FoodNet sites. Clin Infect Dis 38:S285–S296. doi:10.1086/381598 15095201

[8] Taylor EV, Herman KM, Ailes EC, Fitzgerald C, Yoder JS, Mahon BE, Tauxe RV. 2013. Common source outbreaks of *Campylobacter* infection in the USA, 1997–2008. Epidemiol Infect 141:987–996. doi:10.1017/S0950268812001744 PMC9151887 22892294

[9] Ribot EM, Freeman M, Hise KB, Gerner-Smidt P. 2019. PulseNet: entering the age of next-generation sequencing. Foodborne Pathog Dis 16:451–456. doi:10.1089/fpd.2019.2634 PMC6653803 31241352

[10] Bolger AM, Lohse M, Usadel B. 2014. Trimmomatic: a flexible trimmer for Illumina sequence data. Bioinformatics 30:2114–2120. doi:10.1093/bioinformatics/btu170 PMC4103590 24695404

[11] Souvorov A, Agarwala R, Lipman DJ. 2018. SKESA: strategic k-mer extension for scrupulous assemblies. Genome Biol 19:153. doi:10.1186/s13059-018-1540-z PMC6172800 30286803

[12] Gurevich A, Saveliev V, Vyahhi N, Tesler G. 2013. QUAST: quality assessment tool for genome assemblies. Bioinformatics 29:1072–1075. doi:10.1093/bioinformatics/btt086 PMC3624806 23422339

[13] Simão FA, Waterhouse RM, Ioannidis P, Kriventseva EV, Zdobnov EM. 2015. BUSCO: assessing genome assembly and annotation completeness with single-copy orthologs. Bioinformatics 31:3210–3212. doi:10.1093/bioinformatics/btv351 26059717

[14] Li H, Handsaker B, Wysoker A, Fennell T, Ruan J, Homer N, Marth G, Abecasis G, Durbin R, 1000 Genome Project Data Processing Subgroup. 2009. The sequence alignment/map format and SAMtools. Bioinformatics 25:2078–2079. doi:10.1093/bioinformatics/btp352 PMC2723002 19505943

[15] Bortolaia V, Kaas RS, Ruppe E, Roberts MC, Schwarz S, Cattoir V, Philippon A, Allesoe RL, Rebelo AR, Florensa AF, et al.. 2020. ResFinder 4.0 for predictions of phenotypes from genotypes. J Antimicrob Chemother 75:3491–3500. doi:10.1093/jac/dkaa345 PMC7662176 32780112

[16] Camacho C, Coulouris G, Avagyan V, Ma N, Papadopoulos J, Bealer K, Madden TL. 2009. BLAST+: architecture and applications. BMC Bioinformatics 10:421. doi:10.1186/1471-2105-10-421 PMC2803857 20003500

[17] Seemann T. 2020. ABRicate (1.0.1).

[18] Chen L, Zheng D, Liu B, Yang J, Jin Q. 2016. VFDB 2016: hierarchical and refined dataset for big data analysis—10 years on. Nucleic Acids Res 44:D694–D697. doi:10.1093/nar/gkv1239 PMC4702877 26578559

[19] The European Committee on Antimicrobial Susceptibility Testing. 2021. Breakpoint tables for interpretation of MICs and zone diameters. Version 11.0. The European Committee on Antimicrobial Susceptibility Testing (EUCAST). Available from: https://www.eucast.org/fileadmin/src/media/PDFs/EUCAST_files/Breakpoint_tables/v_11.0_Breakpoint_Tables.pdf

[20] Benjamini Y, Hochberg Y. 1995. Controlling the false discovery rate: a practical and powerful approach to multiple testing. Journal of the Royal Statistical Society Series B 57:289–300. doi:10.1111/j.2517-6161.1995.tb02031.x

[21] RStudio Team. 2020. RStudio (4.1.2). Boston, MA.

[22] Yao H, Liu D, Wang Y, Zhang Q, Shen Z. 2017. High prevalence and predominance of the *aph(2″)-If* gene conferring aminoglycoside resistance in *Campylobacter*. Antimicrob Agents Chemother 61:e00112-17. doi:10.1128/AAC.00112-17 PMC5404564 28264854

[23] Lara-Tejero M, Galán JE. 2001. CdtA, CdtB, and CdtC form a tripartite complex that is required for cytolethal distending toxin activity. Infect Immun 69:4358–4365. doi:10.1128/IAI.69.7.4358-4365.2001 PMC98507 11401974

[24] Gu J, Lin Y, Wang Z, Pan Q, Cai G, He Q, Xu X, Cai X. 2022. *Campylobacter jejuni* cytolethal distending toxin induces GSDME-dependent pyroptosis in colonic epithelial cells. Front Cell Infect Microbiol 12:853204. doi:10.3389/fcimb.2022.853204 PMC9093597 35573789

[25] Abuoun M, Manning G, Cawthraw SA, Ridley A, Ahmed IH, Wassenaar TM, Newell DG. 2005. Cytolethal distending toxin (CDT)-negative *Campylobacter jejuni* strains and anti-CDT neutralizing antibodies are induced during human infection but not during colonization in chickens. Infect Immun 73:3053–3062. doi:10.1128/IAI.73.5.3053-3062.2005 PMC1087314 15845513

[26] Guirado P, Iglesias-Torrens Y, Miró E, Navarro F, Attolini CS-O, Balsalobre C, Madrid C. 2022. Host-associated variability of the cdtABC operon, coding for the cytolethal distending toxin, in *Campylobacter jejuni*. Zoonoses Public Health 69:966–977. doi:10.1111/zph.12994 PMC9826217 36053024

[27] Talukder KA, Aslam M, Islam Z, Azmi IJ, Dutta DK, Hossain S, Nur-E-Kamal A, Nair GB, Cravioto A, Sack DA, Endtz HP. 2008. Prevalence of virulence genes and cytolethal distending toxin production in *Campylobacter jejuni* isolates from diarrheal patients in Bangladesh. J Clin Microbiol 46:1485–1488. doi:10.1128/JCM.01912-07 PMC2292937 18287317

[28] Ripabelli G, Tamburro M, Minelli F, Leone A, Sammarco ML. 2010. Prevalence of virulence-associated genes and cytolethal distending toxin production in *Campylobacter* spp. isolated in Italy. Comp Immunol Microbiol Infect Dis 33:355–364. doi:10.1016/j.cimid.2008.12.001 19195703

[29] Panzenhagen P, Portes AB, Dos Santos AMP, Duque S da S, Conte Junior CA. 2021. The distribution of *Campylobacter jejuni* virulence genes in genomes worldwide derived from the NCBI pathogen detection database. Genes (Basel) 12:1538. doi:10.3390/genes12101538 PMC8535712 34680933

[30] Guerry P. 2007. *Campylobacter flagella*: not just for motility. Trends Microbiol 15:456–461. doi:10.1016/j.tim.2007.09.006 17920274

[31] Lertsethtakarn P, Ottemann KM, Hendrixson DR. 2011. Motility and chemotaxis in *Campylobacter* and *Helicobacter*. Annu Rev Microbiol 65:389–410. doi:10.1146/annurev-micro-090110-102908 PMC6238628 21939377

[32] Wassenaar TM, van der Zeijst BA, Ayling R, Newell DG. 1993. Colonization of chicks by motility mutants of *Campylobacter jejuni* demonstrates the importance of flagellin A expression. J Gen Microbiol 139 Pt 6:1171–1175. doi:10.1099/00221287-139-6-1171 8360610

[33] Hendrixson DR, DiRita VJ. 2004. Identification of *Campylobacter jejuni* genes involved in commensal colonization of the chick gastrointestinal tract. Mol Microbiol 52:471–484. doi:10.1111/j.1365-2958.2004.03988.x 15066034

[34] Fearnhead P, Smith NGC, Barrigas M, Fox A, French N. 2005. Analysis of recombination in *Campylobacter jejuni* from MLST population data. J Mol Evol 61:333–340. doi:10.1007/s00239-004-0316-0 16044246

[35] Wysok B, Wojtacka J, Kivistö R. 2020. Pathogenicity of *Campylobacter* strains of poultry and human origin from Poland. Int J Food Microbiol 334:108830. doi:10.1016/j.ijfoodmicro.2020.108830 32841810

[36] González-Hein G, Huaracán B, García P, Figueroa G. 2013. Prevalence of virulence genes in strains of *Campylobacter jejuni* isolated from human, bovine and broiler. Braz J Microbiol 44:1223–1229. doi:10.1590/S1517-83822013000400028 PMC3958191 24688515

[37] Wieczorek K, Wołkowicz T, Osek J. 2018. Antimicrobial resistance and virulence-associated traits of *Campylobacter jejuni* isolated from poultry food chain and humans with diarrhea. Front Microbiol 9:1508. doi:10.3389/fmicb.2018.01508 PMC6039573 30022977

[38] Kordinas V, Nicolaou C, Ioannidis A, Papavasileiou E, Legakis NJ, Chatzipanagiotou S. 2005. Prevalence of four virulence genes in *Campylobacter jejuni* determined by PCR and sequence analysis. Molecular Diagnosis 9:211–215. doi:10.1007/BF03260094 16392901

[39] Iovine NM. 2013. Resistance mechanisms in *Campylobacter jejuni*. Virulence 4:230–240. doi:10.4161/viru.23753 PMC3711981 23406779

[40] Piddock LJV, Ricci V, Pumbwe L, Everett MJ, Griggs DJ. 2003. Fluoroquinolone resistance in *Campylobacter* species from man and animals: detection of mutations in topoisomerase genes. J Antimicrob Chemother 51:19–26. doi:10.1093/jac/dkg033 12493783

[41] Payot S, Bolla J-M, Corcoran D, Fanning S, Mégraud F, Zhang Q. 2006. Mechanisms of fluoroquinolone and macrolide resistance in *Campylobacte*r spp. Microbes Infect 8:1967–1971. doi:10.1016/j.micinf.2005.12.032 16713726

[42] Engberg J, Aarestrup FM, Taylor DE, Gerner-Smidt P, Nachamkin I. 2001. Quinolone and macrolide resistance in *Campylobacter jejuni* and *C. coli*: resistance mechanisms and trends in human isolates. Emerg Infect Dis 7:24–34. doi:10.3201/eid0701.010104 PMC2631682 11266291

[43] Luo N, Pereira S, Sahin O, Lin J, Huang S, Michel L, Zhang Q. 2005. Enhanced *in vivo* fitness of fluoroquinolone-resistant *Campylobacter jejuni* in the absence of antibiotic selection pressure. Proc Natl Acad Sci USA 102:541–546. doi:10.1073/pnas.0408966102 PMC545549 15634738

[44] Dahl LG, Joensen KG, Østerlund MT, Kiil K, Nielsen EM. 2021. Prediction of antimicrobial resistance in clinical *Campylobacter jejuni* isolates from whole-genome sequencing data. Eur J Clin Microbiol Infect Dis 40:673–682. doi:10.1007/s10096-020-04043-y PMC7979593 32974772

[45] Tang Y, Fang L, Xu C, Zhang Q. 2017. Antibiotic resistance trends and mechanisms in the foodborne pathogen, *Campylobacter*. Anim Health Res Rev 18:87–98. doi:10.1017/S1466252317000135 29166961

[46] Acheson D, Allos BM. 2001. *Campylobacter jejuni* infections: update on emerging issues and trends. Clinical Infectious Diseases 32:1201–1206. doi:10.1086/319760 11283810

[47] Cobo-Díaz JF, González del Río P, Álvarez-Ordóñez A. 2021. Whole resistome analysis in *Campylobacter jejuni* and *C. coli* genomes available in public repositories. Front Microbiol 12:662144. doi:10.3389/fmicb.2021.662144 PMC8287256 34290678

[48] Aksomaitiene J, Novoslavskij A, Malakauskas M. 2023. Whole-genome sequencing-based profiling of antimicrobial resistance genes and core-genome multilocus sequence typing of *Campylobacter jejuni* from different sources in Lithuania. Int J Mol Sci 24:16017. doi:10.3390/ijms242116017 PMC10648690 37958998

[49] Deblais L, Jang H, Kauffman M, Gangiredla J, Sawyer M, Basa S, Poelstra JW, Babu US, Harrison LM, Hiett KL, Balan KV, Rajashekara G. 2023. Whole genome characterization of thermophilic *Campylobacter* species isolated from dairy manure in small specialty crop farms of Northeast Ohio. Front Microbiol 14:1074548. doi:10.3389/fmicb.2023.1074548 PMC10071015 37025625

[50] Casagrande Proietti P, Guelfi G, Bellucci S, De Luca S, Di Gregorio S, Pieramati C, Franciosini MP. 2020. Beta-lactam resistance in *Campylobacter coli* and *Campylobacter jejuni* chicken isolates and the association between blaOXA-61 gene expression and the action of β-lactamase inhibitors. Vet Microbiol 241:108553. doi:10.1016/j.vetmic.2019.108553 31928700

[51] Obeng AS, Rickard H, Sexton M, Pang Y, Peng H, Barton M. 2012. Antimicrobial susceptibilities and resistance genes in *Campylobacter* strains isolated from poultry and pigs in Australia. J Appl Microbiol 113:294–307. doi:10.1111/j.1365-2672.2012.05354.x 22672511

[52] Zeng X, Brown S, Gillespie B, Lin J. 2014. A single nucleotide in the promoter region modulates the expression of the β-lactamase OXA-61 in *Campylobacter jejuni*. J Antimicrob Chemother 69:1215–1223. doi:10.1093/jac/dkt515 24408987

[53] Marotta F, Garofolo G, di Marcantonio L, Di Serafino G, Neri D, Romantini R, Sacchini L, Alessiani A, Di Donato G, Nuvoloni R, Janowicz A, Di Giannatale E. 2019. Antimicrobial resistance genotypes and phenotypes of *Campylobacter jejuni* isolated in Italy from humans, birds from wild and urban habitats, and poultry. PLoS One 14:e0223804. doi:10.1371/journal.pone.0223804 PMC6788699 31603950

[54] Hizlisoy H, Sagiroglu P, Barel M, Dishan A, Gungor C, Koskeroglu K, Hizlisoy S, Atalay MA. 2023. *Campylobacter jejuni* and *Campylobacter coli* in human stool samples: antibiotic resistance profiles, putative virulence determinants and molecular characterization of the isolates. World J Microbiol Biotechnol 39:353. doi:10.1007/s11274-023-03786-y 37874390

[55] Quino W, Caro-Castro J, Hurtado V, Flores-León D, Gonzalez-Escalona N, Gavilan RG. 2021. Genomic analysis and antimicrobial resistance of *Campylobacter jejuni* and *Campylobacter coli* in Peru. Front Microbiol 12:802404. doi:10.3389/fmicb.2021.802404 PMC8787162 35087501

[56] Palmieri C, Magi G, Mingoia M, Bagnarelli P, Ripa S, Varaldo PE, Facinelli B. 2012. Characterization of a *Streptococcus* suis *tet*(O/W/32/O)-carrying element transferable to major streptococcal pathogens. Antimicrob Agents Chemother 56:4697–4702. doi:10.1128/AAC.00629-12 PMC3421841 22710115

[57] Warburton PJ, Amodeo N, Roberts AP. 2016. Mosaic tetracycline resistance genes encoding ribosomal protection proteins. J Antimicrob Chemother 71:3333–3339. doi:10.1093/jac/dkw304 PMC5181394 27494928

[58] Ricotta EE, Palmer A, Wymore K, Clogher P, Oosmanally N, Robinson T, Lathrop S, Karr J, Hatch J, Dunn J, Ryan P, Blythe D. 2014. Epidemiology and antimicrobial resistance of international travel-associated *Campylobacter* infections in the United States, 2005–2011. Am J Public Health 104:e108–e114. doi:10.2105/AJPH.2013.301867 PMC4056211 24832415

[59] CDC. 2022. COVID-19: U.S. Impact on antimicrobial resistance, special report 2022. U.S. Department of Health and Human Services, Atlanta, GA. https://www.cdc.gov/drugresistance/covid19.html.

[60] Lin J, Michel LO, Zhang Q. 2002. CmeABC functions as a multidrug efflux system in *Campylobacter jejuni*. Antimicrob Agents Chemother 46:2124–2131. doi:10.1128/AAC.46.7.2124-2131.2002 PMC127319 12069964

[61] Sharifi S, Bakhshi B, Najar-Peerayeh S. 2021. Significant contribution of the CmeABC Efflux pump in high-level resistance to ciprofloxacin and tetracycline in *Campylobacter jejuni* and *Campylobacter coli* clinical isolates. Ann Clin Microbiol Antimicrob 20:36. doi:10.1186/s12941-021-00439-6 PMC8138983 34016127

[62] Kim JM, Hong J, Bae W, Koo HC, Kim SH, Park YH. 2010. Prevalence, antibiograms, and transferable *tet*(O) plasmid of *Campylobacter jejuni* and *Campylobacter coli* isolated from raw chicken, pork, and human clinical cases in Korea. J Food Prot 73:1430–1437. doi:10.4315/0362-028x-73.8.1430 20819352

[63] Dasti JI, Groß U, Pohl S, Lugert R, Weig M, Schmidt-Ott R. 2007. Role of the plasmid-encoded *tet*(O) gene in tetracycline-resistant clinical isolates of *Campylobacter jejuni* and *Campylobacter coli*. J Med Microbiol 56:833–837. doi:10.1099/jmm.0.47103-0 17510271

[64] Ocejo M, Oporto B, Lavín JL, Hurtado A. 2021. Whole genome-based characterisation of antimicrobial resistance and genetic diversity in *Campylobacter jejuni* and *Campylobacter coli* from ruminants. Sci Rep 11:8998. doi:10.1038/s41598-021-88318-0 PMC8076188 33903652

[65] Painset A, Day M, Doumith M, Rigby J, Jenkins C, Grant K, Dallman TJ, Godbole G, Swift C. 2020. Comparison of phenotypic and WGS-derived antimicrobial resistance profiles of *Campylobacter jejuni* and *Campylobacter coli* isolated from cases of diarrhoeal disease in England and Wales, 2015–16. J Antimicrob Chemother 75:883–889. doi:10.1093/jac/dkz539 31943013

[66] Frazão MR, Cao G, Medeiros MIC, Duque S da S, Allard MW, Falcão JP. 2021. Antimicrobial resistance profiles and phylogenetic analysis of *Campylobacter jejuni* strains isolated in Brazil by whole genome sequencing. Microb Drug Resist 27:660–669. doi:10.1089/mdr.2020.0184 33021437

[67] FDA. 2023. The national antimicrobial resistance monitoring system: enteric bacteria methods. U.S. Food and Drug Administration. Available from: https://www.fda.gov/media/101741/download?attachment

